# Association between body roundness index and advanced cardiovascular-kidney-metabolic syndrome

**DOI:** 10.3389/fnut.2025.1623766

**Published:** 2025-07-30

**Authors:** Yuehui Chen, Hongxiang Tu, Min Liang, Mo Shen

**Affiliations:** Department of Clinical Laboratory, Key Laboratory of Clinical Laboratory Diagnosis and Translational Research of Zhejiang Province, The First Affiliated Hospital of Wenzhou Medical University, Wenzhou, China

**Keywords:** body roundness index, CKM, NHANES, obesity, RCS

## Abstract

**Background:**

Cardiovascular–kidney–metabolic (CKM) syndrome is a disease characterized by interrelated cardiovascular, renal, and metabolic abnormalities. The body roundness index (BRI) is an innovative anthropometric index that characterizes human body shape by calculating body roundness and using eccentricity to estimate visceral and total body fat percentages. The present study aimed to clarify the relation between BRI and advanced CKM syndrome.

**Methods:**

Data were obtained from the National Health and Nutrition Examination Survey conducted between 2005 and 2018. Tertiles based on data analysis were applied to divide the levels of BRI. Regression methods assessed the association of BRI with the risk of advanced CKM syndrome. Moreover, receiver operating characteristic analysis determined BRI’s predictive performance for such a syndrome.

**Results:**

The present study included 12,329 participants, with an advanced CKM occurrence rate of 12.20%. Regression analysis revealed a positive association between BRI and advanced CKM syndrome. After adjusting for covariates, individuals in the highest BRI tertile (T3) showed a significantly higher prevalence of advanced CKM syndrome compared to the reference group (odds ratio: 1.49; 95% confidence interval: 1.09–2.03, *p* < 0.001). Smoothed curve-fitting analyses showed a linear trend. Subgroup analyses yielded results consistent with the total results. Furthermore, restricted cubic spline analyses indicated that BRI was a stronger predictor of advanced CKM syndrome than other anthropometric measures.

**Conclusion:**

An increased BRI is associated with a higher occurrence rate of advanced CKM syndrome. BRI demonstrated a stronger correlation with advanced CKM than traditional measures and warrants consideration as a preferred metric for CKM risk stratification.

## 1 Introduction

Epidemiological studies have identified cardiovascular disease (CVD), diabetes mellitus (DM), and chronic kidney disease (CKD) as significant interrelated health issues that collectively contribute to increased mortality rates within the population (1, 2). The American Heart Association (AHA), in October 2023, introduced the concept of cardiovascular–kidney–metabolic (CKM) syndrome as a disorder that encompasses the interactions between CVD, metabolic risk factors, and CKD (3). This syndrome is associated with elevated rates of multi-organ dysfunction and harmful cardiovascular effects. The complex pathophysiological links between these diseases involve key factors such as inflammation, oxidative stress, vascular dysfunction, and insulin resistance (4, 5). An increasing volume of research has supported this concept, and pathological studies have demonstrated that these disorders frequently coexist in patients (6). CKM syndrome is systematically categorized into stages from 0 (no risk factors) to 4 (confirmed CVD) to adequately describe its evolving nature, with stages 3 and 4 referred to as advanced CKM syndrome.

Obesity constitutes a significant global health threat and serves as a risk factor for type 2 DM, hypertension, and CVD (7). The incidence of obesity in adults continues to rise in many countries, with over 1.9 billion individuals (39% of the global population) classified as overweight by 2023, of which 650 million meet the criteria for obesity (8). Although body mass index (BMI) is the primary measure to assess obesity, it cannot accurately reflect adipose tissue distribution. In 2013, Thomas DM et al. (9) introduced a new body roundness index (BRI) to estimate the share of visceral fat relative to overall body fat by integrating waist circumference (WC) and height. Compared to BMI, this method can more precisely evaluate abdominal fat accumulation, particularly visceral fat, thereby enhancing the prediction of related disease risks. BRI is related to diabetes and CVD (10–17). However, the relationship of BRI with CKM syndrome has not been analyzed in the US population. This study aimed to explore the relationship between BRI and the probability of developing advanced CKM in US adults. The study is expected to generate novel insights and information that will inform future prevention and diagnostic strategies for CKM syndrome.

## 2 Materials and methods

### 2.1 Study design and objects

The study used data from the National Health and Nutrition Examination Survey (NHANES), a program conducted by the Centers for Disease Control and Prevention (CDC). Approval for the survey protocol was granted by the CDC, with informed consent secured from all individuals involved. The complete dataset from the NHANES study is publicly accessible via https://www.cdc.gov/nchs/nhanes/.

**Figure 1:**
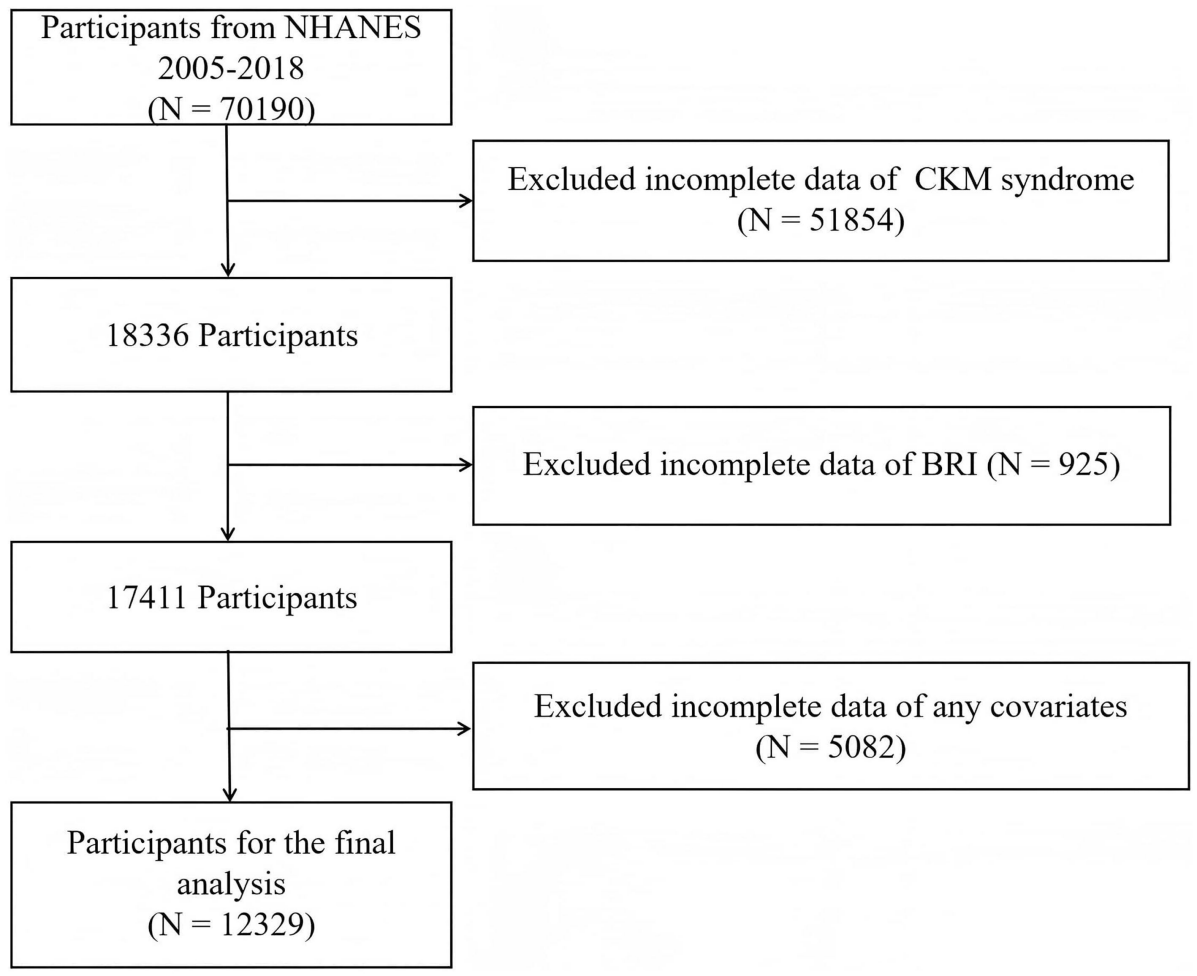
Flow diagram of the study cohort selection.

This research used data from 2005 to 2018. The inclusion criteria were as follows: individuals who provided information through questionnaires, interview transcripts, and laboratory tests. The initial study population comprised 70,190 participants. Individuals who lacked data regarding CKM syndrome, BRI, or covariates were excluded, resulting in 12,329 eligible participants. The participant screening process is shown in Figure 1.

### 2.2 BRI measurement

The BRI served as the indicator, which can be determined via the standardized formula: BRI = 364.2–365.5 × (1 – [WC (m)/2 π ]^2^/[0.5 × height (m)]^2^)^½^ (9). The survey protocol received approval from the CDC, and informed consent was obtained from all participants. Height and WC data were derived from the patients’ physical examination records. For subsequent models, BRI was stratified into three groups based on tertiles, with the T1 group serving as the reference.

### 2.3 Ascertainment of the stages of CKM syndrome

The different stages of CKM syndrome (ranging from 0 to 4) were defined according to the 2023 AHA guidelines (4). Stages included: Stage 0: individuals with no metabolic abnormalities and in perfect health; Stage 1: individuals characterized by overweight, obesity, or adipose tissue dysfunction; Stage 2: individuals with moderate and high risk (per KDIGO and AHA guidelines); Stage 3: individuals at high risk or with a 10-year cardiovascular risk (≥20%); and Stage 4: individuals with diagnosed CVD or end-stage organ damage. Advanced CKM syndrome encompasses stages 3 and 4, as individuals in these stages have confirmed CVD or face a significant risk of experiencing cardiovascular events in the short term (18). A description of the staged assessment can be found in Supplementary Table S1.

### 2.4 Covariates

The demographic data were gathered using structured questionnaires, including age, gender, ethnic background, educational level, poverty–income ratio (PIR), and marital status. In addition, personal behavioral variables, such as tobacco use and alcohol intake, were included. Hypertension and DM were identified through indicator measurements, medication use, and self-reports. Potential confounding factors were considered in the physical and laboratory assessments, including BMI, WC, blood pressure measurements, lipid indicators, creatinine and uric acid serum levels, and glycohemoglobin. Detailed definitions are provided in Supplementary Table S2.

### 2.5 Statistical analysis

NHANES applied a stratified, multistage probability sampling design to oversample specific populations. The basic data were adjusted with NHANES examination weights to address unequal sampling probabilities and enhance data accuracy. In this study, continuous variables were examined by weighted one-way analysis of variance. The data were exhibited as means with standard errors. The weighted 2 test was used to analyze the categorical variables and presented in the form of frequency (percentage). To analyze the relation of BRI with advanced CKM syndrome, a three-stage weighted multifactor logistic regression model was constructed: Model 1 was the unadjusted crude model, while Model 2 was corrected for demographic variables and Model 3 was further based on Model 2 to include BMI, PIR, HDL-C, TC, serum creatinine, smoking status, drinking status, serum uric acid, glycated hemoglobin, and blood pressure measurements. Moreover, the dose–response relationship between BRI and advanced CKM syndrome was verified by restricted cubic spline (RCS) analysis. The study further conducted stratified analyses to test for subgroup heterogeneity by age, gender, education, BMI, smoking status, diabetes, and hypertension, and assessed intergroup differences by interaction *p*-values. Finally, the predictive efficacy of WC/ BMI/ BRI for advanced CKM syndrome was systematically evaluated using the receiver operating characteristic (ROC) curves and the area under the curve (AUC). All analyses were conducted using R 4.3.1, and *p*-values <0.05 were set as the threshold for significance.

## 3 Results

### 3.1 Baseline features of the study population

Up to 12,329 individuals were classified by BRI tertiles, as detailed in Table 1. The BRI values <4.27 were grouped into the first tertile (T1), 4.27–6.02 to T2, and > 6.02 to T3. The average prevalence of advanced CKM syndrome was 12.20%, with an ascending trend across the BRI tertiles (T1, 5.82%; T2, 12.97%; T3, 19.05%; *p* < 0.001). Compared to the BRI T1 group, the patients in the T3 group were mostly women, older, Mexican American, less educated, and had a low PIR. They had a higher probability of being former smokers, non-drinkers, or former drinkers and of experiencing conditions such as diabetes and hypertension. For biochemical markers, the BRI T3 group showed increases in several markers, such as serum uric acid, TC, and glycohemoglobin, while HDL-C levels were lower. In addition, physiological parameters such as SBP, DBP, BMI, and WC were also higher in the BRI T3 group compared to the T1 group.

**Table 1:** Weighted characteristics of the study population according to the tertiles of BRI^a^.

Characteristics	Total	T1 (<4.27)	T2 (4.27–6.02)	T3 (>6.02)	*p*-value
Number	12,329	4,111	4,108	4,110	
Age (years)	47.05 (0.28)	41.21 (0.38)	49.45 (0.32)	51.54 (0.39)	< 0.001
Sex (%)					< 0.001
Male	6,215 (49.87)	2,210 (51.32)	2,318 (56.03)	1,687 (41.61)	
Female	6,114 (50.13)	1901 (48.68)	1790 (43.97)	2,423 (58.39)	
Ethnicity (%)					< 0.001
Non-Hispanic White	5,620 (69.69)	1934 (70.43)	1806 (69.18)	1880 (69.34)	
Non-Hispanic Black	2,374 (10.35)	810 (10.43)	706 (9.04)	858 (11.65)	
Mexican American	1914 (8.02)	409 (5.42)	759 (9.65)	746 (9.43)	
Other ethnicities	2,421 (11.94)	958 (13.72)	837 (12.14)	626 (9.59)	
Educational attainment (%)					< 0.001
High school or less	5,653 (38.10)	1,536 (31.49)	1979 (39.61)	2,138 (44.44)	
More than high school	6,676 (61.90)	2,575 (68.51)	2,129 (60.39)	1972 (55.56)	
Marital status (%)					< 0.001
Married or living with a partner	7,533 (64.54)	2,368 (61.31)	2,713 (69.02)	2,452 (63.70)	
Living alone	4,796 (35.46)	1743 (38.69)	1,395 (30.98)	1,658 (36.30)	
Smoking status (%)					< 0.001
Never	6,734 (54.43)	2,323 (56.54)	2,215 (53.51)	2,196 (52.87)	
Now	2,495 (20.03)	984 (23.35)	795 (19.32)	716 (16.79)	
Former	3,100 (25.54)	804 (20.11)	1,098 (27.17)	1,198 (30.34)	
Drinking status (%)					< 0.001
Never	1,620 (10.30)	457 (8.77)	529 (10.14)	634 (12.30)	
Mild	4,330 (37.79)	1,585 (39.52)	1,460 (39.12)	1,285 (34.31)	
Moderate	1887 (17.47)	713 (19.60)	603 (15.77)	571 (16.72)	
Heavy	2,512 (21.39)	924 (23.89)	852 (21.94)	736 (17.82)	
Former	1980 (13.04)	432 (8.22)	664 (13.02)	884 (18.86)	
Hypertension (%)					< 0.001
No	7,138 (62.72)	3,140 (80.11)	2,337 (60.93)	1,661 (43.73)	
Yes	5,191 (37.28)	971 (19.89)	1771 (39.07)	2,449 (56.27)	
DM (%)					< 0.001
No	9,796 (84.82)	3,827 (95.58)	3,318 (86.67)	2,651 (69.93)	
Yes	2,533 (15.18)	284 (4.42)	790 (13.33)	1,459 (30.07)	
Advanced CKM syndrome (%)					< 0.001
No	10,262 (87.80)	3,753 (94.18)	3,377 (87.03)	3,132 (80.95)	
Yes	2067 (12.20)	358 (5.82)	731 (12.97)	978 (19.05)	
PIR	3.04 (0.04)	3.19 (0.05)	3.11 (0.04)	2.80 (0.05)	< 0.001
BMI (kg/m^2^)	28.96 (0.10)	23.22 (0.06)	28.45 (0.06)	36.38 (0.14)	< 0.001
WC (cm)	99.18 (0.26)	83.93 (0.16)	99.27 (0.14)	117.39 (0.28)	< 0.001
HDL-C (mg/dL)	54.21 (0.24)	60.06 (0.34)	52.42 (0.37)	49.09 (0.30)	< 0.001
TC (mg/dL)	193.09 (0.59)	188.18 (0.73)	198.27 (0.84)	193.52 (1.07)	< 0.001
Serum creatinine (mg/dL)	0.88 (0.00)	0.88 (0.01)	0.90 (0.01)	0.87 (0.01)	< 0.001
Serum uric acid (mg/dL)	5.50 (0.02)	5.05 (0.03)	5.59 (0.03)	5.93 (0.03)	< 0.001
Glycohemoglobin (%)	5.60 (0.01)	5.32 (0.01)	5.58 (0.01)	5.94 (0.03)	< 0.001
DBP (mmHg)	69.83 (0.21)	68.02 (0.25)	70.43 (0.29)	71.36 (0.28)	< 0.001
SBP (mmHg)	120.99 (0.24)	116.05 (0.32)	121.99 (0.33)	125.88 (0.37)	< 0.001

T, tertiles; BRI, body roundness index; CKM, cardiovascular kidney metabolic; DM, diabetes mellitus; PIR, poverty income ratio; BMI, body mass index; WC, waist circumference; HDL-C, high-density lipoprotein cholesterol; TC, total cholesterol; DBP, diastolic blood pressure; SBP, systolic blood pressure.

a Values are weighted means (standardized errors) or number of participants (weighted percentages) unless otherwise indicated.

### 3.2 Association of BRI with advanced CKM syndrome risk

**Figure 2:**
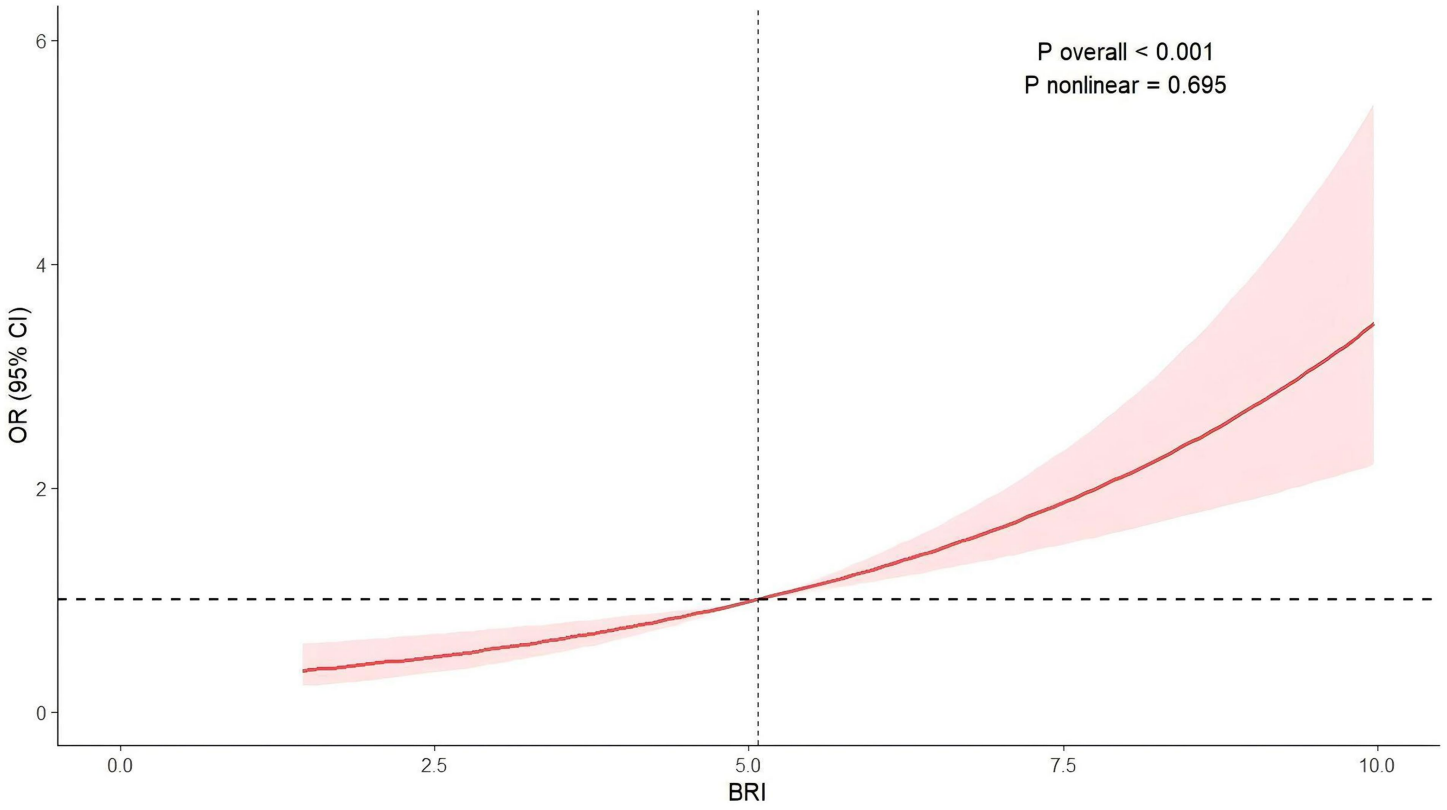
Association between BRI and advanced CKM syndrome. Adjustment factors included age, gender, ethnicity, educational attainment, marital status, BMI, PIR, smoking status, drinking status, HDL-C, TC, serum creatinine, serum uric acid, glycohemoglobin, SBP, and DBP.

The relation of BRI with advanced CKM syndrome was still consistent in every weighted regression mode (Table 2; Supplementary Figure S1). Model 3 demonstrated a positive association between BRI and advanced CKM syndrome (odds ratio (OR): 1.29, 95% confidence interval (CI): 1.16–1.43, *p* < 0.001). For sensitivity analysis, we categorized the BRI and re-examined its correlation with outcomes. In Model 3, individuals in the BRI T3 group demonstrated a greater risk compared to the BRI T1 group (OR: 1.49, 95% CI: 1.09–2.03, *p* = 0.013). These results were consistent with partially adjusted models. Furthermore, our study analyzed in depth the potential dose–response relation of BRI with advanced CKM syndrome risk based on RCS regression analysis (Figure 2). The findings showed a linear relationship in Model 3 (*p* for non-linear = 0.695). More specifically, our analysis reveals a positive correlation between BRI elevation and advanced CKM syndrome risk. This dose–response relationship demonstrates progressive risk intensification, where each unit increase in BRI corresponds to disproportionately higher ORs at upper quantiles. Notably, we observed a non-threshold effect: even minimal BRI increments were associated with risk elevation.

**Table 2:** Weighted multivariate logistic regression analysis of BRI and advanced CKM syndrome^a^.

Characteristic	Model 1 OR (95% CI), *p*-value	Model 2 OR (95% CI), *p*-value	Model 3 OR (95% CI), *p*-value
BRI (continuous)	1.20 (1.17, 1.23), <0.001	1.17 (1.13, 1.22), <0.001	1.29 (1.16, 1.43), <0.001
BRI (categorical)			
T1 (<4.27)	Reference	Reference	Reference
T2 (4.27–6.02)	2.41 (2.04, 2.85), <0.001	1.33 (1.11, 1.59), 0.002	1.22 (0.98, 1.51), 0.079
T3 (>6.02)	3.81 (3.20, 4.54), <0.001	2.11 (1.74, 2.57), <0.001	1.49 (1.09, 2.03), 0.013
*p* for trend	<0.001	<0.001	0.013

T, tertiles; BRI, body roundness index; CKM, cardiovascular kidney metabolic; PIR, poverty income ratio; BMI, body mass index; HDL-C, high-density lipoprotein cholesterol; TC, total cholesterol; DBP, diastolic blood pressure; SBP, systolic blood pressure; CI, confidence interval; OR, odds ratios.

a Model 1: unadjusted; Model 2: adjusted for age, gender, ethnicity, educational attainment, and marital status; Model 3: adjusted for age, gender, ethnicity, educational attainment, marital status, BMI, PIR, smoking status, drinking status, HDL-C, TC, serum creatinine, serum uric acid, glycohemoglobin, SBP, and DBP.

### 3.3 Subgroup analysis

**Figure 3:**
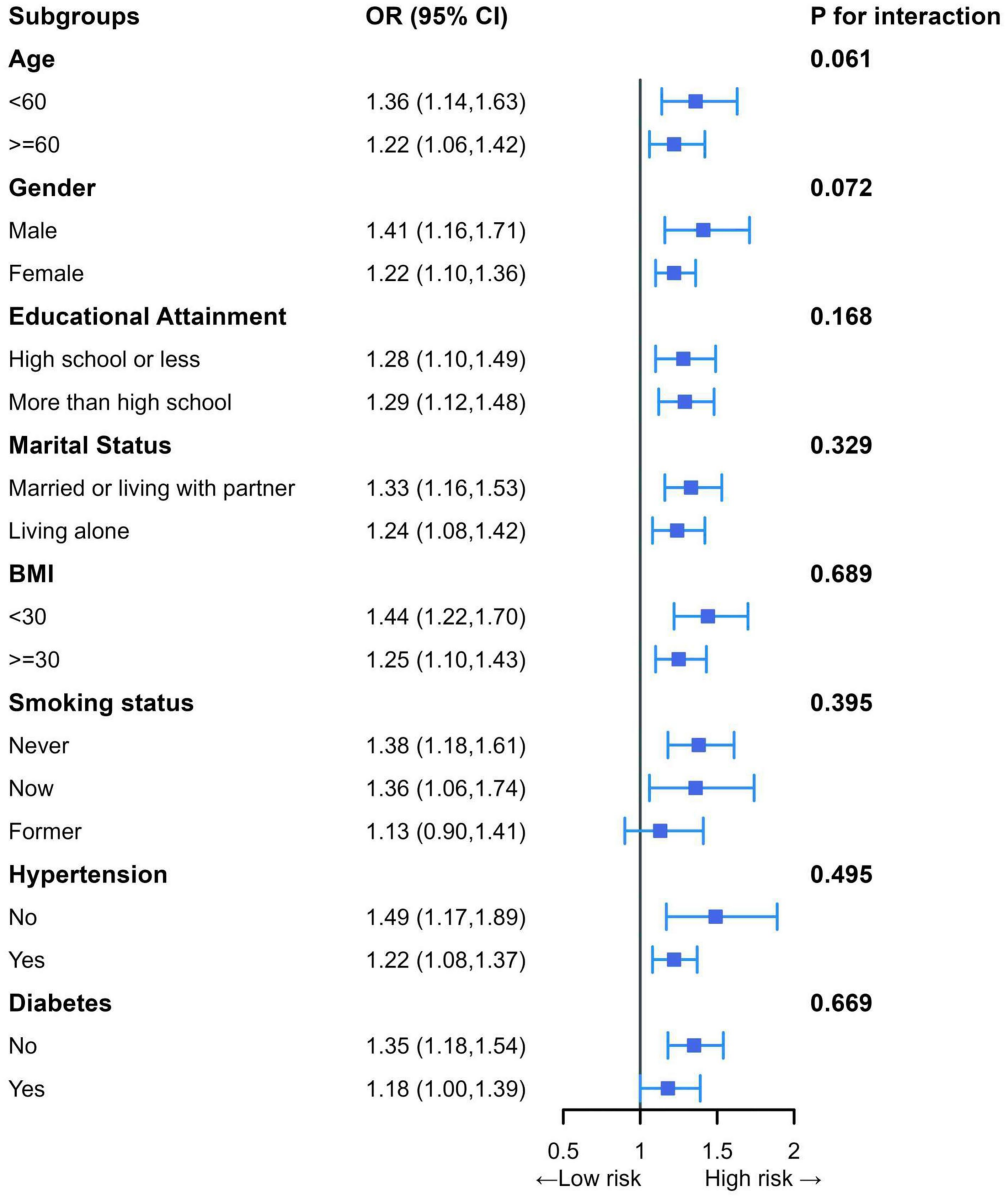
Subgroup analyses of the association between BRI and advanced CKM syndrome. Adjusted for age, sex, ethnicity, educational attainment, marital status, BMI, PIR, smoking status, drinking status, HDL-C, TC, serum creatinine, serum uric acid, glycohemoglobin, SBP, and DBP.

Subgroup analyses examining the association between BRI and advanced CKM syndrome showed that none of the interaction terms were statistically significant (Figure 3). This suggests that their relationship existed in all subgroups, similar to that in the general population.

### 3.4 Comparing WC, BMI, and BRI as predictors of advanced CKM syndrome

**Figure 4:**
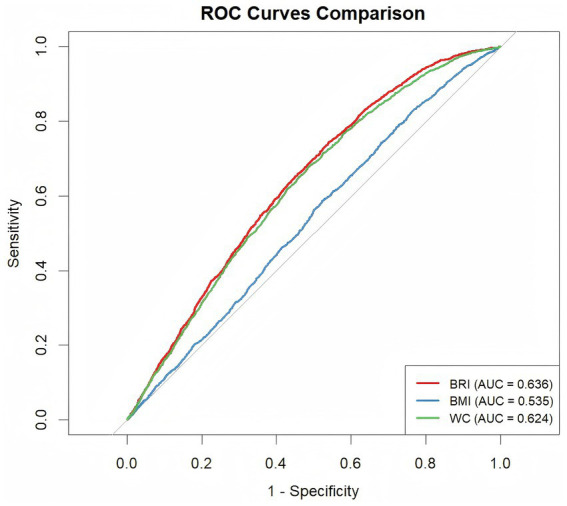
Receiver operating characteristic curves of the BRI, BMI, and WC for the risk of advanced CKM syndrome.

According to the ROC analysis (Table 3; Figure 4), BRI exhibited the highest performance (AUC: 0.636) for predicting advanced CKM syndrome, followed by WC (0.624) and BMI (0.535). Furthermore, BRI had a sensitivity of 74.4% and a specificity of 45.9%, WC had that of 67.6 and 51.7%, respectively, and BMI had that of 58.7 and 47.7%. DeLong’s test suggested that the BRI outperformed both BMI and WC in predicting advanced CKM syndrome (*p* < 0.001 for both comparisons).

**Table 3:** AUC of the BRI, BMI, and WC for the risk of advanced CKM syndrome.

Anthropometric measures	AUC (95% CI)	Cutoff	Sensitivity	Specificity	PPV	NPV	DeLong’s test
BRI	0.636 (0.623–0.648)	4.736	0.744	0.459	0.217	0.899	Reference
BMI	0.535 (0.522–0.548)	27.525	0.587	0.477	0.184	0.851	<0.001
WC	0.624 (0.612–0.637)	97.450	0.676	0.517	0.22	0.888	<0.001

CKM, cardiovascular kidney metabolic; BRI, body roundness index; BMI, body mass index; WC, waist circumference; AUC, area under the curve; CI, confidence interval; PPV, positive predictive value; NPV, negative predictive value.

## 4 Discussion

An analysis of 12,329 NHANES participants indicated the association between increased BRI levels and advanced CKM syndrome in Americans. This relation was also pronounced even after adjustments, which indicated that advanced CKM syndrome may be influenced by BRI; each one-unit enhancement in BRI was related to a 29% enhancement in the odds of having advanced CKM syndrome. Individuals in the uppermost BRI category (T3) showed an elevated probability of developing advanced CKM syndrome than the T1 group. This finding was further corroborated by the linear nature of the association of BRI with advanced CKM syndrome, as demonstrated in the present study. It has to be pointed out that even minimal BRI increments were associated with risk elevation, indicating the absence of a biologically safe BRI threshold. Furthermore, subgroup analyses demonstrated that these associations were maintained across diverse populations. In addition, BRI demonstrated the highest predictive value and the largest AUC for advanced CKM syndrome compared to BMI and WC. Consequently, these findings provide compelling evidence that the increased BRI is an important risk factor for advanced CKM syndrome.

A key contributor to CKM syndrome and the CVD epidemic is obesity (6). The advantage of the BRI over traditional body measurements is that the ratio of visceral adipose tissue can be accurately estimated, providing a clear understanding of fat distribution. By assessing the risk of obesity based on WC, the BRI provided a more in-depth assessment of fat distribution conditions. As a result, it could more accurately evaluate the risk of CKD, CVD, and DM than traditional indicators such as BMI and WC. BRI was strongly associated with metabolic equivalent scores (MetS) relative to BMI and waist-to-height ratio (WtHR) (19, 20). Li′s team analyzed for the first time the relation between BRI and MetS in a US population and showed that BRI was better than other indicators, including WC (11). Qiu et al. suggested that higher BRI was positively associated with DM risk in US adults and that BRI had a better predictive value than BMI, ABSI, and WC (21). Moreover, He et al. found that a higher BRI was related to a heightened risk of CVD in the US population, and BRI can also precisely predict CVD and congestive heart failure as WC, but it is superior to BMI (22). Obesity is a typical risk factor that increases the risk of CKD and progression to severe renal disease (23–26). Zhang et al. reported that BRI was independently related to a higher occurrence rate of CKD among obese adults in the US (27).

Antonio’s team analyzed the prevalence of abdominal obesity (AO), high WtHR, and excess adiposity (EA) as well as their association with CKM syndrome in a survey of 6,588 Spanish adults. The findings revealed that AO, WtHR, and EA were good indicators for evaluating obesity and that abdominal obesity was associated with CKM syndrome (28). A nationwide longitudinal study in China showed that AO, indicated by BRI, could be a valuable indicator of frailty progression, especially in the onset of CKM. A high BRI, as well as advanced CKM progression, further increased the risk of frailty. Notably, the predictive power of BRI is superior to that of BMI (29).

While there exist many potential mechanisms for the relationship between CKM symptoms, it has been proposed that the syndrome primarily stems from excess and/or dysfunctional adipose tissue (30). Adipose tissue secretes abundant pro-inflammatory factors and oxidative stress products, triggering systemic inflammatory responses and oxidative stress. These factors not only damage vascular endothelial cells but also promote the development of atherosclerosis, which increases the risk of CVD (31). Furthermore, they may also aggravate insulin resistance. In addition, obesity-induced metabolic disorders and hemodynamic changes can cause direct and indirect damage to the kidneys. These pathophysiological mechanisms are a significant factor in the advancement of CVD and CKD (4). However, the association of BRI with advanced CKM has not been clarified in the United States; therefore, the present study validated the existence of a linear association of BRI with advanced CKM syndrome in US adults, which had not been found in earlier studies. This study found that BRI is an effective indicator in predicting advanced CKM syndrome. In addition, a positive relation of BRI with advanced CKM was consistently found in subgroup analyses, highlighting the robustness of the finding.

It has to be noted that the criteria for CKM syndrome stages vary across studies based on data availability. American Heart Association (AHA) defines: Stage 0, which represents no CKM risk factors; Stage 1, excess or dysfunctional adiposity; Stage 2, metabolic risk factors or moderate to high-risk chronic kidney disease; Stage 3, subclinical CVD in CKM, or risk equivalents of subclinical CVD (high-risk CKD or high predicted risk of CVD); and Stage 4, clinical CVD with CKM risk factors (4). Nevertheless, cardiac biomarkers required for its implementation, specifically high-sensitivity troponin, were unavailable in NHANES 2005–2018, while recent guidelines encourage PREVENT criteria (32). Consequently, we adopted the CKM staging methodology from a JAMA Network Open study on social determinants of health (33), an approach validated by subsequent investigations (34, 35). Moreover, we revised CKM staging criteria to explicitly account for racial/ethnic differences, incorporating methodological refinements based on another CKM study (18). However, the current staging definition exhibits notable limitations, particularly for Stage 3 classification. While we referenced multiple established criteria, the NHANES dataset lacks key diagnostic parameters, including coronary artery calcification scores, echocardiographic data, and advanced cardiac biomarkers. Consequently, we were constrained to use the PREVENT score cutoff of 20% as a surrogate measure, despite its inability to fully capture the complex cardiometabolic pathophysiology inherent in CKM syndrome. These methodological constraints highlight the need for future validation studies using enhanced PREVENT score frameworks with contemporary risk factors and multimodal diagnostic approaches to improve staging accuracy in American populations.

This study possessed two notable strengths. The data were analyzed reliably, and the sample size was sufficiently large to accurately represent the present situation in the US population. Additionally, adjustments for confounding covariates and the consistent association of BRI with advanced CKM syndrome across subgroup analyses underscored the result’s robustness. Despite these strengths, several limitations were evident. First, due to its cross-sectional design, the study could not analyze the causal relationship between BRI and the development of advanced CKM syndrome. Second, while potential confounders were considered in the data analysis, residual confounding could not be entirely excluded. Consequently, the operational definitions of CKM stages were contingent upon data availability. For instance, using a PREVENT cut-off of 20% is effectively “de-risking” many patients who previously were high risk based on the pooled cohort equation, but more accurately calibrating to their true risk. Finally, due to the limitations of the database, the findings may not be generalizable to populations beyond the United States.

## 5 Conclusion

Overall, there is a positive relationship between increased BRI and advanced CKM syndrome. Further analyses showed that BRI outperformed BMI and WC in predicting advanced CKM syndrome, making it a useful tool for early assessment and clinical decision-making.

## Data Availability

Publicly available datasets were analyzed in this study. This data can be found here: https://www.cdc.gov/nchs/nhanes/.
