# Supplementary material for: Association between body roundness index and advanced cardiovascular-kidney-metabolic syndrome
Source: Front Nutr. 2025 Jul 30;12:1623766. doi: 10.3389/fnut.2025.1623766 (PMC12343269; doi:10.3389/fnut.2025.1623766)
Supplement: Supplementary file 1 [file Table_1.docx]

**Supplementary Table S1. Assessment of CKM Syndrome Stages**

| **Stage 0** | 1. Normal BMI (<23 kg/m² for Asians; <25 kg/m² for other groups) 2. Normal waist circumference (<80 cm for Asian women; <90 cm for Asian men; <88 cm for other women; <102 cm for other men) 3. No criteria met for other stages |
| --- | --- |
| **Stage 1** | Individuals with any of the following:   1. Elevated BMI (≥23 kg/m² for Asians; ≥25 kg/m² for others) 2. Elevated waist circumference (≥80 cm for Asian women; ≥90 cm for Asian men; ≥88 cm for other women; ≥102 cm for other men) 3. Prediabetes (glycohemoglobin 5.7%–6.4% or fasting glucose 100–125 mg/dL) |
| **Stage 2** | Individuals with moderate-to-high-risk CKD (per KDIGO and AHA guidelines) or metabolic risk factors.  Metabolic risk factors include elevated fasting triglycerides (≥135 mg/dL), hypertension, diabetes, or metabolic syndrome (≥3 of the following: elevated waist circumference, low HDL [<40 mg/dL for men, <50 mg/dL for women], fasting triglycerides ≥150 mg/dL, elevated blood pressure [systolic ≥130 mmHg, diastolic ≥80 mmHg, or antihypertensive use], or prediabetes). |
| **Stage 3** | Individuals with:   1. Very-high-risk CKD (per KDIGO criteria) 2. High-predicted 10-year cardiovascular risk (≥20%), estimated using the AHA PREVENT equations. |
| **Stage 4** | Individuals with self-reported, established cardiovascular disease, including any of the following: coronary heart disease, angina, heart attack, heart failure, or stroke. |

The absence of cardiac biomarkers, cardiovascular imaging, and data on atrial fibrillation and peripheral artery disease is due to database limitations, which restrict the identification of subclinical CVD and the accuracy of staging. Additionally, reliance on self-reported data introduces biases, compromising the reliability of cardiovascular condition classification.

**Supplementary Table S2. The detail definition and classification of covariates.**

| **Variables** | **Definitions or Classification** |
| --- | --- |
| Sex | Male, Female. |
| Race | Non-Hispanic White, Non-Hispanic Black, Mexican American, Other Race. |
| Education attainment | High school or less, More than high school. |
| Marital status | Married or living with partner, Living alone. |
| Smoking status | Never smoking: <100 cigarettes in lifetime;  Former smoking: >100 cigarettes in life and smoke not at all now;  Current smoking: >100 cigarettes in lifetime. |
| Drinking status | Never: consumed fewer than 12 drinks in their lifetime;  Former: consumed at least 12 drinks in one year but did not drink in the last year or did not drink in the last year but had at least 12 drinks in their lifetime;  Heavy: consumed at least 3 drinks per day for females, at least 4 drinks per day for males, or engaged in binge drinking on 5 or more days per month;  Moderate: consumed at least 2 drinks per day for females, at least 3 drinks per day for males, or engaged in binge drinking on at least 2 days per month;  Mild : consumed at most 1 drink per day for females, at most 2 drinks per day for males. |
| Hypertension | An average systolic blood pressure (SBP) equal to or exceeding 140 mmHg;  An average diastolic blood pressure (DBP) equal to or exceeding 90 mmHg;  Self-reported hypertension;  Individuals taking prescribed anti-hypertensive medications. |
| DM | i) physician confirmation of diabetes diagnosis, ii) glycohemoglobin levels equal to or greater than 6.5%, iii) fasting glucose ≥ 7.0 mmol/L, iv) random blood glucose≥ 11.1 mmol/L, and v) documented use of DM medication. |
| BMI | weight (kg)/height (m^2^). |
| Laboratory tests | The Specific method can be found in this webpage  ([https://wwwn.cdc.gov/nchs/nhanes/continuousnhanes/labmethods.aspx?BeginYear=200](https://wwwn.cdc.gov/nchs/nhanes/continuousnhanes/labmethods.aspx?BeginYear=2017)5) |

Abbreviations: DM, diabetes mellitus; BMI, body mass index.

**
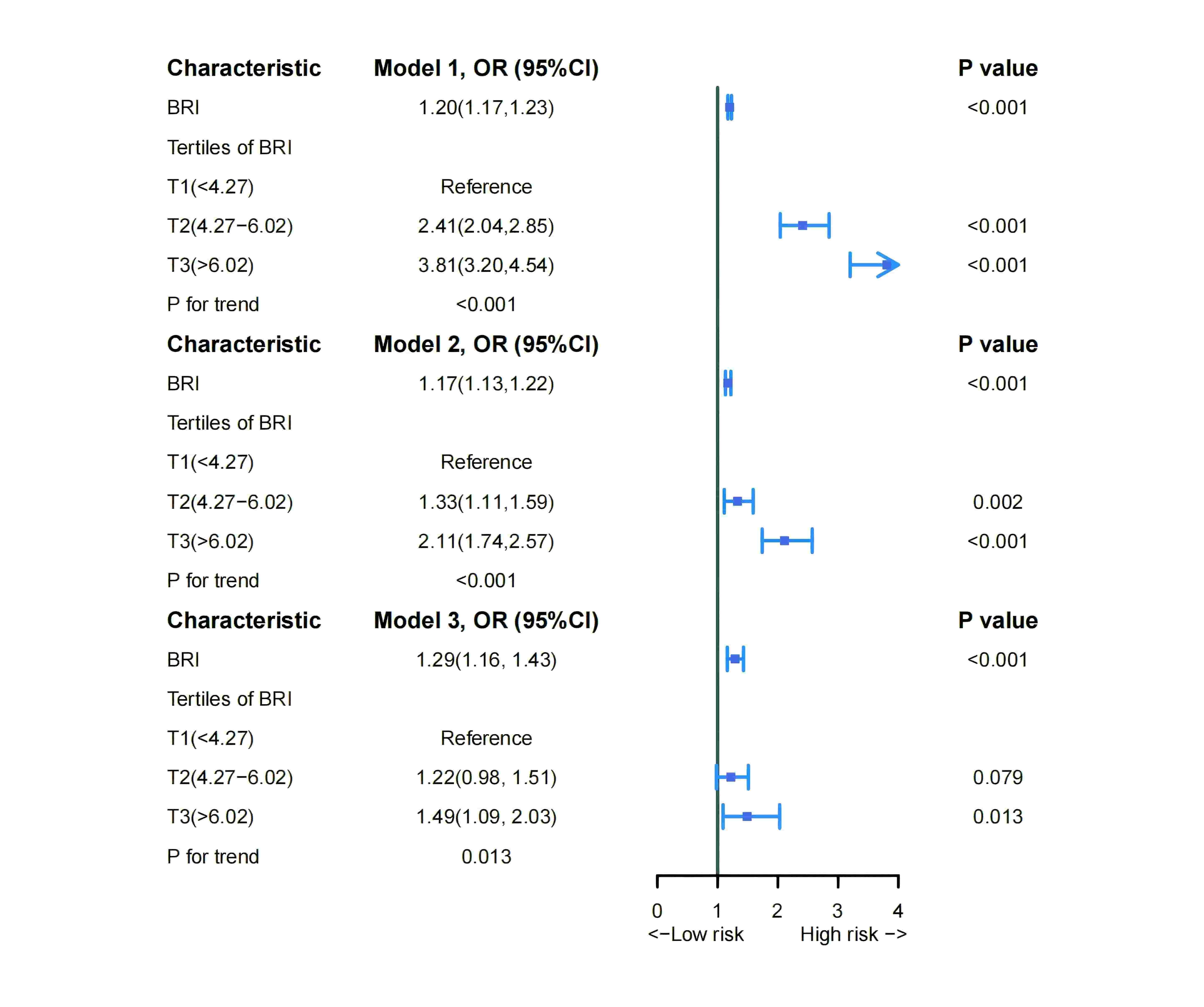
**

**Supplementary figure S1. Weighted multivariate logistic regression analysis of BRI and advanced CKM syndrome**^a^

^a^Model 1: unadjusted; Model 2: adjusted for age, sex, race, educational attainment, and marital status; Model 3: adjusted for age, sex, race, education attainment, marital status, BMI, PIR, smoking status, drinking status, HDL-C, TC, serum creatinine, serum uric acid, glycohemoglobin, SBP, and DBP.
